# Supplementary material for: Cross-modality mapping using image varifolds to align tissue-scale atlases to molecular-scale measures with application to 2D brain sections
Source: Nat Commun. 2024 Apr 25;15:3530. doi: 10.1038/s41467-024-47883-4 (PMC11045777; doi:10.1038/s41467-024-47883-4)
Supplement: Supplementary file 1 — Supplementary Information [file 41467_2024_47883_MOESM1_ESM.pdf]

Supplementary Information for Cross-Modality Mapping using Image Varifolds to Align Tissue-Scale Atlases to Molecular-Scale Measures with Application to 2D Brain Sections

Kaitlin M. Stouffer*[1,2,3], Alain Trouvé[3], Laurent Younes[4], Michael Kunst[5], Lydia Ng[5], Hongkui Zeng[5], Manjari Anant[1], Jean Fan[1], Yongsoo Kim[6], Xiaoyin Chen[5], Mara Rue[5], and Michael I. Miller*[1,2]

[1]Department of Biomedical Engineering, Johns Hopkins University, Baltimore, MD, USA
[2]Kavli Neuroscience Discovery Institute, Johns Hopkins University, Baltimore, MD, USA
[3]Centre Borelli, ENS Paris-Saclay, Gif-sur-yvette, France
[4]Department of Applied Mathematics and Statistics, Johns Hopkins University, Baltimore, MD, USA
[5]Allen Institute for Brain Science, Seattle, WA, USA
[6]Department of Neural and Behavioral Sciences, Penn State University, College of Medicine, State College, PA, USA

* Correspondance to Kaitlin M. Stouffer (kstouff4@jhmi.edu) and Michael I. Miller (mim@jhu.edu)

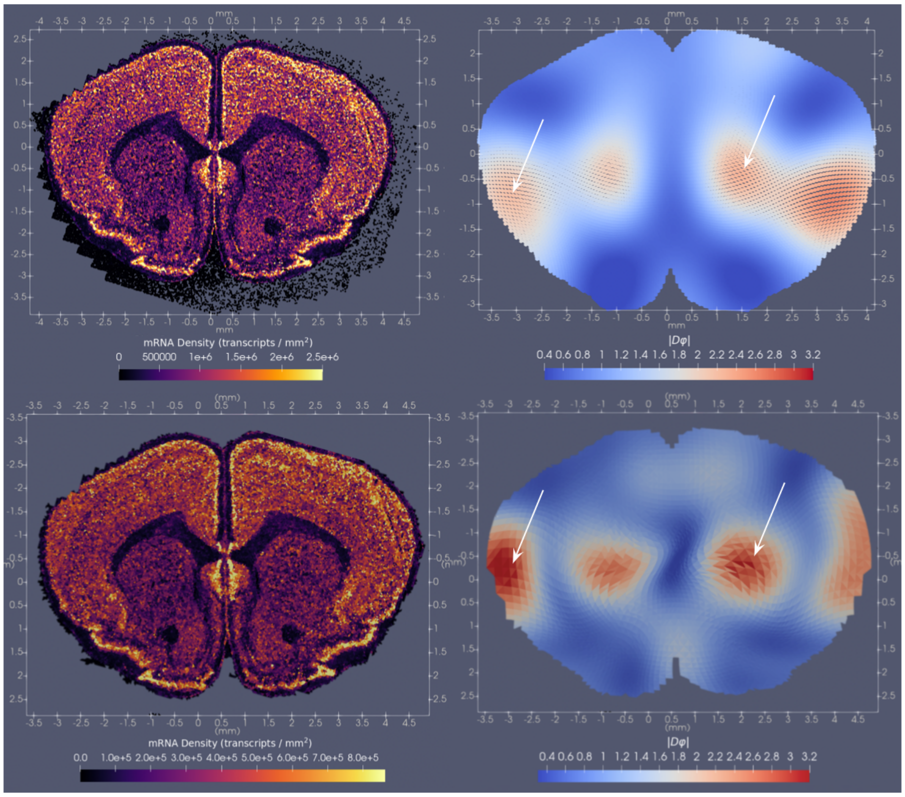

Supplementary Fig. 1: Comparable renderings at 50 μm resolution of MERFISH cell-independent transcriptomics section and corresponding geometric mapping of CCFv3 section $Z = 385$ out of 1320 to transcriptomics section. Top shows rendering of initial section as point cloud with $63k$ particles, with estimated determinant of the Jacobian of diffeomorphic mapping of CCFv3 section to transcriptomics. Bottom shows rendering as mesh with $15k$ simplices and estimated determinant of the Jacobian of diffeomorphic mapping of CCFV3 section to transcriptomics with similar regions of expansion and contraction.

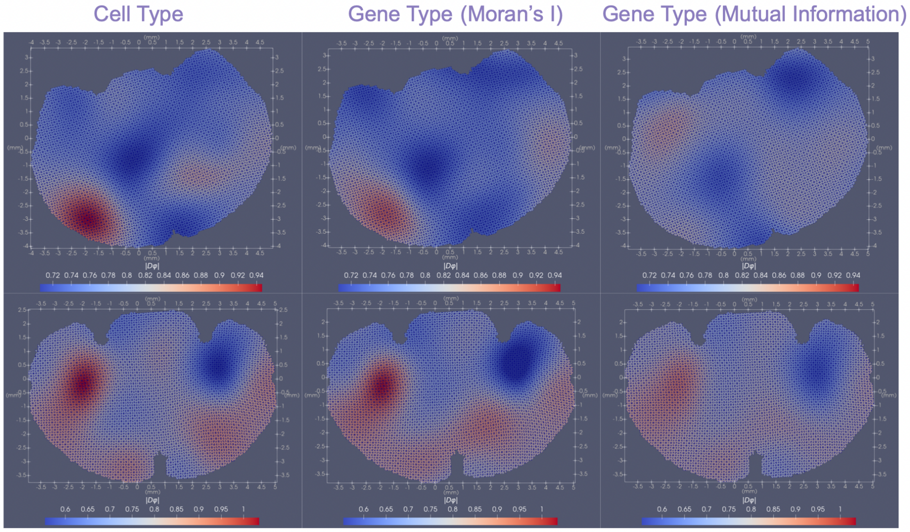

Supplementary Fig. 2: Similarity in diffeomorphisms estimated to map CCFv3 sections to the same MERFISH sections carrying different features. Diffeomorphism taking CCFv3 section $Z = 675$ (top) and $Z = 890$ (bottom) out of 1320 to corresponding MERFISH sections shown in Figures 3 and 6, respectively, estimated in tandem with three separate latent distributions over varying feature spaces (33 cell types, 6 gene types with high Moran's I index, 7 gene types with high spatial mutual information). Determinant of the Jacobian exhibits areas of expansion (red) and contraction (blue) in all three settings, with similar patterns across feature spaces.

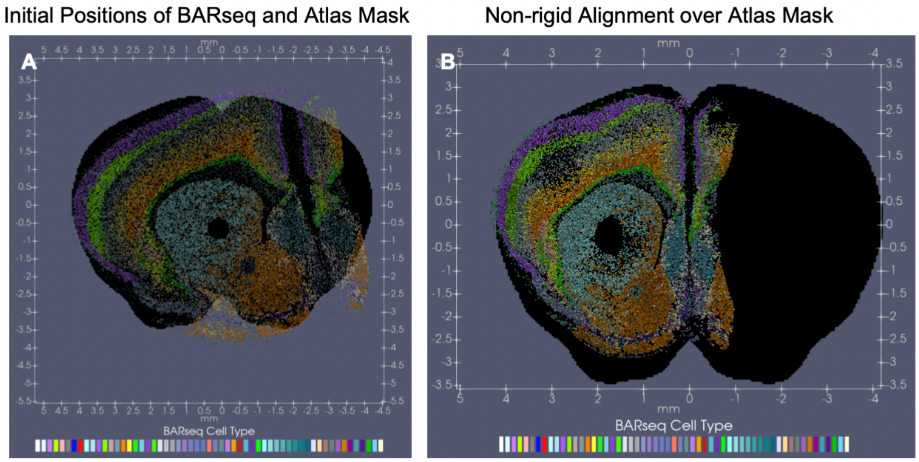

Supplementary Fig. 3: Mapping CCFv3 section $Z = 437$ out of 1320 to BARseq cell-typed partial coronal section. A shows initial positioning of CCFv3 and BARseq sections, with CCFv3 section depicted as black mask. B shows alignment after pulling back BARseq onto CCFv3 section via inverse estimated diffeomorphism (φ^{-1}).

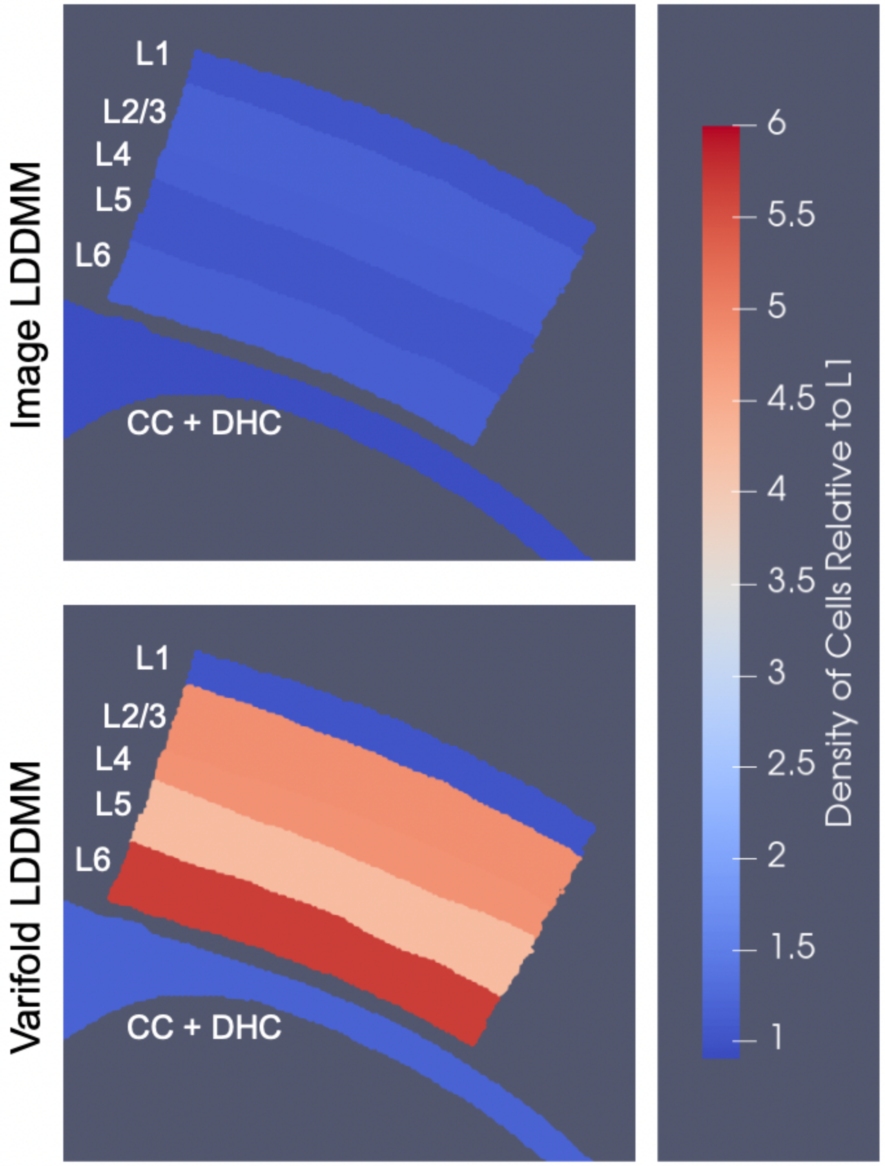

Supplementary Fig. 4: Comparative cell density in layers of cortex and white matter (corpus callosum and dorsal hippocampal commissure (CC+DHC)) for BARseq cell-segmented coronal section mapped to CCFv3 section $Z = 837$ via estimation of diffeomorphism using image-based LDDMM (top) versus image-varifold based LDDMM (bottom). Densities per region computed as cells per square millimeter of atlas tissue in each respective region. For comparison between methods, densities in each region are normalized against the absolute density in layer 1 (L1). xIV-LDDMM yields expected cell densities 4-6x higher in layers 2-6 compared with L1 and CC+DHC versus LDDMM, where similar cell densities across cortical layers and white matter result from misalignment in these areas.

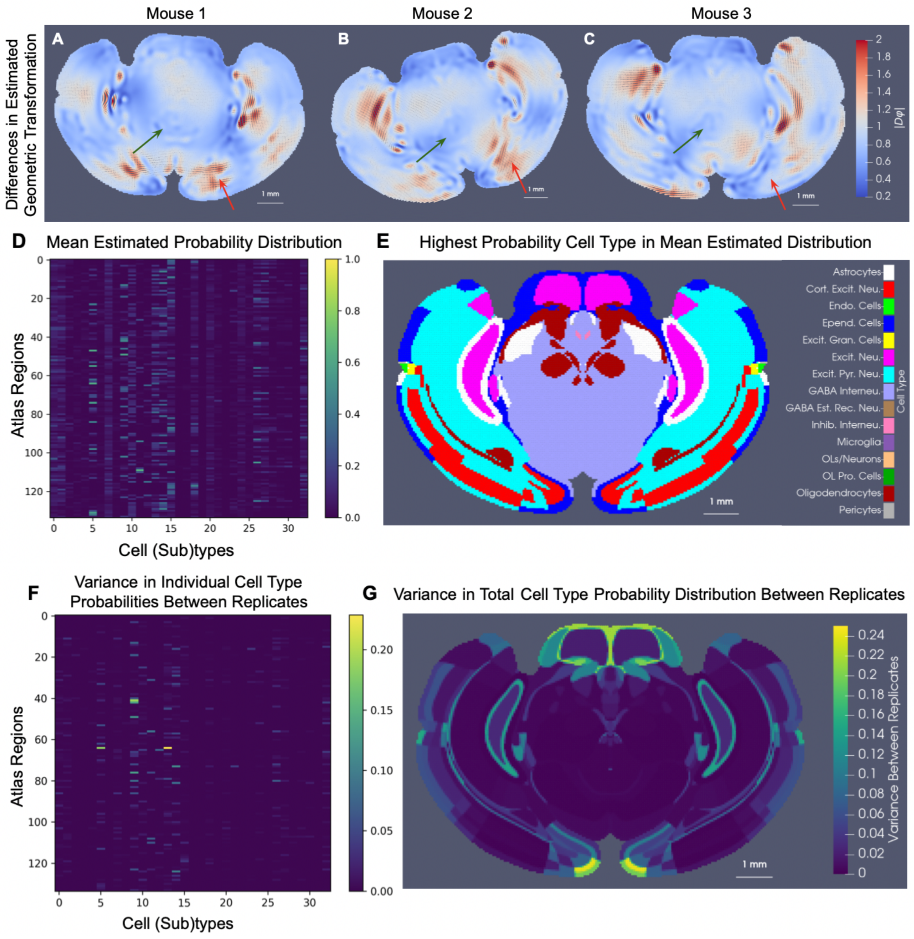

Supplementary Fig. 5: Comparison of geometry and cell type distribution in 3 MERFISH replicates via estimated geometric transformations (φ) and cell type distributions (π) estimated with xIV-LDDMM. A-C show determinant of the jacobian for estimated diffeomorphism taking CCFv3 section to individual MERFISH coordinates. Green arrows show similarities in geometry across replicates via equal amounts of contraction; red arrows indicate differences in geometry via differing levels of contraction/expansion. D-G highlight similarities and differences in estimated cell type distribution across replicates. D shows mean probability distribution over cell types for each CCFv3 region, computed from those estimated in xIV-LDDMM for each replicate. Highest probability cell type in mean distribution per CCFv3 region shown in E. F shows variance in estimated probability per cell type for each CCFv3 across the 3 distributions estimated via xIV-LDDMM in mapping the CCFv3 section to the 3 replicates. G shows the total variance across estimated cell type probabilities between replicates.

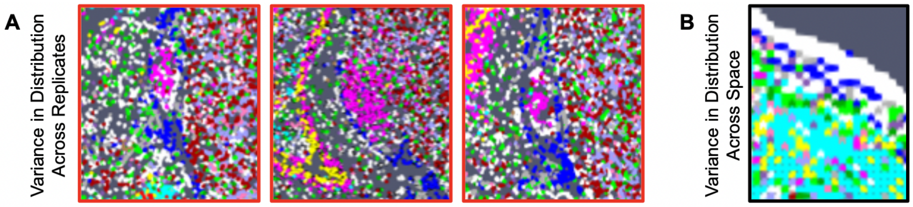

Supplementary Fig. 6: Areas of variance in cell type distribution across replicates (A) and across a single CCFv3 region of space (B). A shows area of the medial geniculate nucleus with varying proportions of excitatory neurons (pink), astrocytes (white), and ependymal cells (blue). B shows postpiriform transition area with outer cortical regions dominated by ependymal cells (blue) versus inner cortical regions dominated by excitatory pyramidal neurons (cyan).

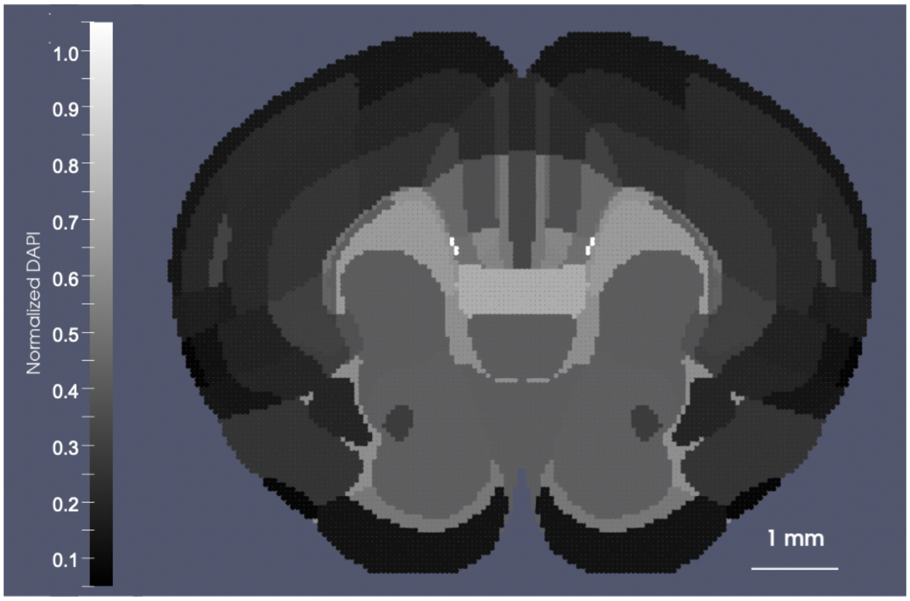

Supplementary Fig. 7: Mean normalized DAPI intensity per CCFv3 region following inverse transformation of DAPI image to CCFv3 coordinates. Foreground pixels only selected by thresholding and mean intensity computed per CCFv3 region based on foreground pixels aligning to within each region.
